# Fabrication and failure characteristics of asymmetric balsa-core based fibre composite sandwich beams under 3-point bending test

**DOI:** 10.1016/j.heliyon.2024.e26502

**Published:** 2024-02-19

**Authors:** Prahlad Kumar, K. Priya Ajit, Jayant Prakash Varun

**Affiliations:** Department of Mechanical Engineering, Indian Institute of Technology (ISM), Dhanbad, India

**Keywords:** Sandwich structure, Composite, Asymmetric balsa-core, Glass fibre loading (GL) face, Carbon fibre loading (CL) face, 3-Point bending

## Abstract

The failure characteristics of an asymmetric balsa-core based fibre composite sandwich beam subjected to 3-point bending are investigated analytically and experimentally. The experimental specimens comprise a balsa wood core and two types of fibre composite skins, notably glass fibre and carbon fibre. During the static bending test, the effects of carbon fibre loading (CL) face and glass fibre loading (GL) face on bending failure behaviour are tested. Since the skin thickness, span lengths, and core thicknesses has substantial effect on the structural failures. Therefore, a detailed analysis has been carried out considering the effect of varying span lengths, skin thicknesses, and core thicknesses on several failure modes, particularly indentation, face yield, and core-shear. For the analysis, fourteen specimens have been fabricated, each with a specific geometry and face loading conditions. This report consists of a detailed fabrication flow and loading conditions. Finally, the work has been benchmarked with already published report on asymmetric sandwich structures. The analysis's predictions and the results of the experiment indicate remarkable concordance.

## 1 Introduction

Sandwich structures’ primary advantages are their high stiffness and strength-to-weight ratios; hence, the idea is advantageous in situations where weight plays a significant role in the design process. The integration of such structures predominates in aerospace engineering, such as aeroplane cabin flooring, control surfaces, landing bay doors, helicopter rotor blades and fuselages, satellite antennas, and solar panels [1]. Sandwich-based, self-supporting truck frames prove to be lighter than traditional metal structures, which results in increasing payload and profitability [2]. Sandwich-designed truck tankers for liquid fuels, milk, juice or other substances have proved to be effective for load-bearing structures, and provides thermal insulation [3]. Sandwich structures have two thin stiff faces and a thick lightweight core [4]. The faces provide the flexural stiffness of the sandwich structures, while the core provides shear strength and interaction between the faces. The two faces are identical when referring to a conventionally symmetric sandwich panel. Materials such as aluminium, steel, and fibre-reinforced polymer are frequently used for the face, while balsa, honeycombs, trusses, and foams are essential for the core [5]. However, employing a lightweight core material makes it possible to significantly enhance the moment of inertia of the cross-section and the overall flexural stiffness and the bearing capacity of the structure [6]; nevertheless, this will not result in a considerable increase in the total weight of the structure. Hanifehzadeh et al. [7] studied the response of a steel concrete steel wall when exposed to a blast load in close proximity. The results indicate that the suggested wall structure exhibits reduced out-of-plane distortion and enhanced resistance to blast-induced damage. Kueh et al. [8] focused on the impact behavior of a dual-layer sandwich beam, which has the potential to enhance the impact resistance of structural components. The findings indicate that the arched core mitigates the stress that leads to failure, which is usually concentrated at the impact location in all of the scenarios analysed. The flexural and buckling characteristics of sandwich composites were investigated by Radhakrishnan et al. [9]. The findings indicated that a reduced aspect ratio offered significant benefits in terms of both bending and buckling for the sandwich laminates. Kueh et al. [10] used numerical analysis to study the effectiveness of impact resistance in a newly developed sandwich beam with a laterally arched core. They found that the AISI1018 core-equipped construction has a better impact resistance efficiency index than other samples, making it a promising option for a dual-core sandwich beam structure.

The asymmetric sandwich structures provide a plethora of design alternatives due to their diverse performance characteristics [[11], [12], [13]]. The use of new asymmetric sandwich structures with two faces, distinct in material and shape, has become increasingly popular in past decades. In addition, balsa usage in the engineering sector has received significant attention because it is a lightweight and low-density material that provides high stiffness and strength in the sandwich structure [14].

Over the past few decades, researchers have shown significant interest in determining various failure modes of sandwich beams, starting from the collapse mechanisms under the bending test, as determined by the authors [15], to three and four-point bending tests. Further, Authors [[16], [17], [18]] have presented symmetric sandwich beams by analytically determining the limit load for each failure mode, such as face yield, core shearing, indentation, and face wrinkling (*discussed in the later section*). Farrokhabadi et al. [19] focused on the experimental and numerical analysis of innovative multilayered sandwich panels. The study investigates the influence of integrating a corrugated core on the flexural properties of composite sandwich panels. The result is found by comparing the maximum loads, energy absorption, and damage mechanisms of reinforced specimens (made up of several layers) and non-reinforced specimens. Moreover, Crupi et al. [16] investigated the impact of a distinct failure mechanism on the energy absorption capacity of an aluminium foam core sandwich construction using static and dynamic analysis of symmetric sandwich beams. Hamidin et al. [20] conducted an experimental and numerical analysis on sandwich panels featuring both filled and unfilled sine and square corrugated cores. The findings suggested that the use of polyurethane foam would substantially enhance the capacity to absorb energy and will also lengthen the lifespan of the sandwich panel. Apart from these, very few studies have been conducted on asymmetric sandwich beams, such as Frostig et al. [21] conducted a theoretical investigation on the bending of an asymmetrical sandwich beam that contained a foam-like core. Recently, Qin et al. [22]; Wang et al. [23]; and Qin et al. [24] developed yield criteria for geometrically and physically asymmetric metal sandwich structures, respectively. Further, Zhang et al., [25]; and Zhang et al. [26] have reported that exchanging two faces of asymmetric aluminium foam core sandwich beams with various thicknesses led to significant differences in failure mechanism maps. Later on, Zhang et al. [27] reported that instead of using aluminium, composite faces have shown minimal variation in failure mechanism maps. Moreover, Wang et al. [28] have examined the failure behaviour of asymmetric sandwich beams experimentally as well as theoretically and concluded that when a structurally asymmetric sandwich beam collapses via face yield, the load can constantly grow till the ‘second failure’. On the other hand, the authors [29] have designed, constructed, and tested the GFRP-Balsa sandwich bridge deck. Their findings confirm that the composite sandwich deck meets the requirements for reduced fatigue, high performance, cost-effectiveness, and ultimate limit state, thereby ensuring its serviceability.

Despite several experimental and analytical studies on the failure mechanism of symmetric and asymmetric sandwich beams, there is a dearth of research on integrating balsa wood as a core with a composite face in asymmetric sandwich beams. The main difference between the beam studied in the current study and other sandwich beams containing balsa as a core from the literature is the use of asymmetric fiber composite skins. We have used carbon fiber on one face of the beam and glass fiber on the other face. This is a novel approach that has not been well studied in the literature. The integration of the balsa core to the sandwich beams, especially in the asymmetric sandwich beam, improves the performance by providing higher stiffness and strength to the sandwich structure. Additionally, this approach offers cost savings for the overall structure. Thus, it becomes crucial to investigate the failure modes considering balsa as a core with composite faces.

The present study entails an experimental and analytical investigation of asymmetric sandwich structures considering balsa as the core material and fibre composite as the face material. The reason behind considering balsa-core is to improve efficiency in cost, lightweight, stiffness, strength, or performance. On the other hand, the fibre composite material provides a comparatively higher strength-to-weight ratio because young's modulus of the material is far greater than other reported materials. In addition, the proposed structure is subjected to 3-point bending to determine its failure modes in terms of indentation, face yield, and core shear (*discussed in the later section*). For the analysis, balsa wood was used as the core material of the specimen. In contrast, glass fibre composite and carbon fibre composite are used for the face sheets to make the physically asymmetric structure. The sample is considered physically asymmetric due to the different material on its top and bottom faces. On the top face, there is a bidirectional carbon fiber mat, and on the bottom face, there is a bidirectional glass fiber mat. Moreover, the entire asymmetric structure was integrally created in a mold employing a Vacuum-Assisted Resin Transfer Technique (VARTM).

## 2 Test structure, material details, Fabrication details, and experimental procedure

**Fig. 1:**
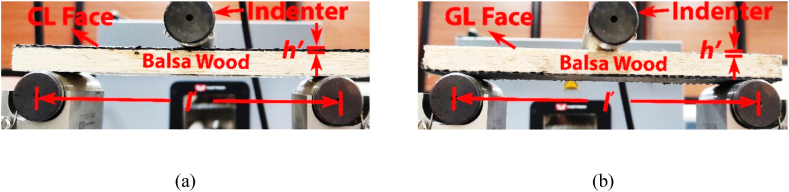
Cross-section view of asymmetric beam before 3-point bending test under: (a) Carbon Loading (CL) face, and (b) Glass Loading (GL) Face.

The cross-section view of the asymmetric sandwich beam prior to 3-point bending (unloaded) has been depicted in Fig. 1. Fig. 1 depicts the placement of carbon fibre reinforced polymer (CFRP) composites in Fig. 1(a) and glass fibre reinforced polymer (GFRP) composites in Fig. 1(b) as the respective face materials for the asymmetric sandwich beams. For the analysis, 1.15 mm, 1.40 mm, and 1.85 mm skin thickness (*hʹ*) of CFRP and GFRP composites are employed. In addition, the core of the structure is made up of balsa wood, where the wood cores are also of three distinct thicknesses (*cʹ*): 9 mm, 12 mm, and 15 mm. Finally, the effect of span lengths (*lʹ*) is analysed for 272 mm, 124 mm, and 107 mm, respectively. Furthermore, to investigate the maximum efficiency of the structure, it underwent both carbon fibre loading (CL) and glass fibre loading (GL) conditions. To carry out the 3-point bending test, two support rollers (bottom rollers) are at a distance of *lʹ* (i.e., span lengths), and load is applied to the top roller (centre of the structure).

*Material Details:* A commercially accessible bidirectional carbon fibre mat of 400 GSM and a glass fibre mat of 400 GSM are procured from C. F. Composites, New Delhi, India. After receiving the product, preventive measures are implemented to prevent any inadvertent external modifications to the mat before its usage. Apart from these, the other relevant materials that are mandatory during the fabrication process, such as release agent, sealant tape, infusion mesh, acetone, vacuum bag, epoxy-resin, and hardener, are also procured from C. F. Composites, New Delhi, India. On the other hand, the balsa wood used for the core material is acquired from Asiatic Enterprise, Mumbai, India.

**Fig. 2:**
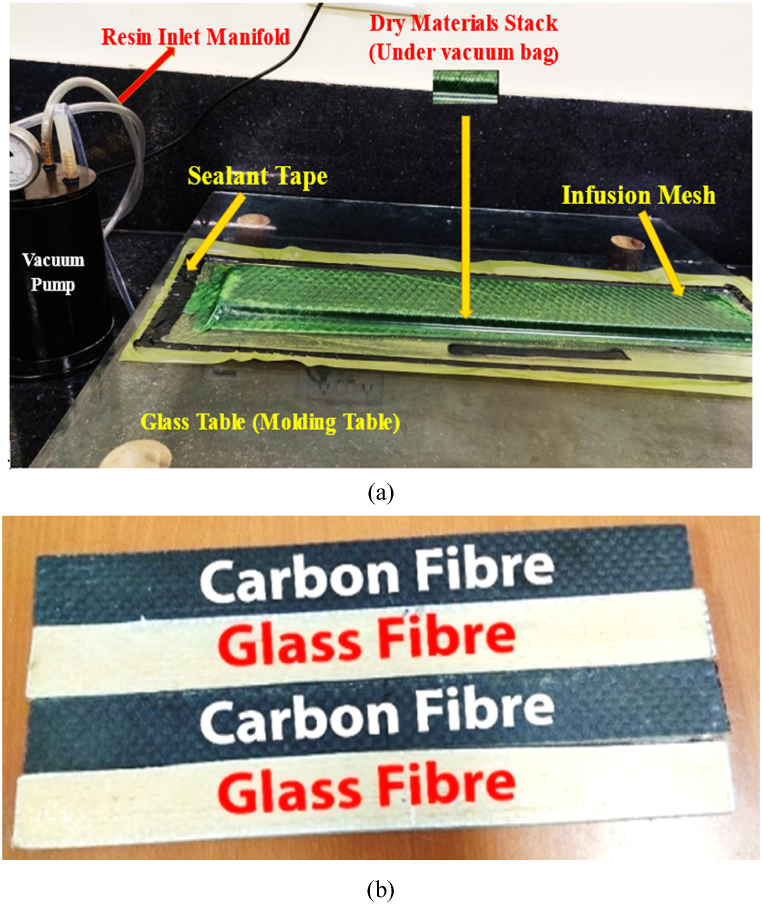
(a) fabrication flow of asymmetric balsa-core fibre composite sandwich beams, and (b) test specimen prior to 3-point static bending test.

*Fabrication Details:* In order to fabricate the asymmetric sandwich beams, a vacuum molding technique (VARTM) based on the film stacking sequence has been employed. This technique results in low-cost with exceptional results with a vast array of distinct varieties, notably fibre-reinforced polymers against the proposed structure. By using film stacking sequence, an asymmetric sandwich beam is fabricated that has different properties on the top and bottom faces. To begin the fabrication, firstly, the workspace of the glass/molding table has been coated with a releasing agent prior to the placement of the fibre mat to facilitate the process of removing the sandwich samples once fabricated, as depicted in Fig. 2(a). Secondly, three layers of bidirectional glass fibre mat were laid, then balsa wood was put over the glass fibre mat, followed by the deposition of three layers of bidirectional carbon fibre mat over the balsa wood, as depicted in Fig. 1(a). Thirdly, the vacuum chamber was prepared with a vacuum bag and sealant tape, as depicted in Fig. 2(a). Later, Epoxy-resin and hardener in a proper ratio were appropriately mixed and then supplied to the vacuum chamber via resin inlet manifold through the vacuum pump, as depicted in Fig. 2(a). In this fabrication process, no additional interfacial material was incorporated between the composite skin and the core. By applying vacuum, VARTM, a closed-molding technique, infuses an epoxy resin mixture into a preform composed of layered fabrics and other materials. After the completion of resin infusion to the vacuum chamber, the sandwich beam was cured for the next 24 h at room temperature. Finally, a diamond saw cutter was used to cut the test specimen in the required dimensions, as depicted in Fig. 2(b). The geometrical dimension of the test specimen employed for the 3-point bending is tabulated in Table 1.

**Table 1:** Geometrical dimension of the asymmetric fibre composite sandwich test specimen.

Specimen Label	h' (*mm)*	c' (*mm*)	l' (*mm*)	l'+2l_H_'(*mm*)	c'/l'	h'/c'	Aspect Ratio (l'/c')
**CL-01**	1.15	15	272	340	0.0551	0.0766	18.13
**GL-02**	1.15	15	272	340	0.0551	0.0766	18.13
**GL-03**	1.15	15	124	155	0.1209	0.0766	8.27
**GL-04**	1.15	15	107	133.75	0.1401	0.0766	7.13
**CL-05**	1.15	15	107	133.75	0.1401	0.0766	7.13
**CL-06**	1.15	15	124	155	0.1209	0.0766	8.27
**GL-09**	1.15	12	272	340	0.0441	0.0958	22.67
**CL-10**	1.15	12	272	340	0.0441	0.0958	22.67
**GL-11**	1.4	09	272	340	0.0330	0.1555	30.22
**CL-12**	1.4	09	272	340	0.0330	0.1555	30.22
**GL-13**	1.4	15	107	133.75	0.0841	0.1555	7.13
**CL-14**	1.4	15	107	133.75	0.0841	0.1555	7.13
**GL-17**	1.85	15	107	133.75	0.1401	0.1233	7.13
**CL-18**	1.85	15	107	133.75	0.1401	0.1233	7.13

**Fig. 3:**
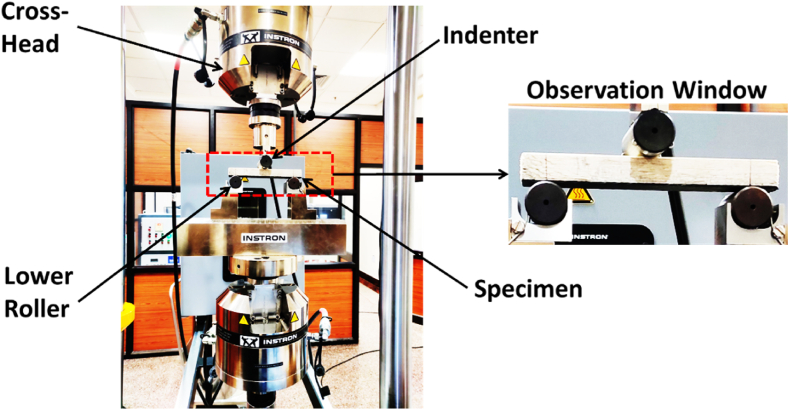
Test bench set-up to perform 3-point bending test.

*Experimental Procedure:* To begin the procedure, the 3-point static bending test is performed in accordance with the standard of ASTM C393-00 (ASTM, 1916), as depicted in Fig. 3. It depicts the test bench set-up to perform a 3-point bending test of the specimen. The observation window as an inset under Fig. 3 depicts the initial loading scenario, particularly the Carbon Loading (CL) of asymmetric balsa-core fibre composite sandwich specimen. To project the load to the test specimen, an Instron Universal Testing Machine (UTM) with a capacity of 100 kN is used. The cross-head displacement offered by the projected load is in terms of 1.5 mm/min*.*

For the sanity of our analysis, the specimen has been considered in such a way that one parameter was varied, keeping the other two parameters constant, as tabulated in Table 1. A monitoring system of the UTM has been used to record the data for load and deflection. On the other hand, real-time pictures of deformation and mode of failures are taken manually. Further, to pin-point the initial mode of failure, the highest peak of load versus deflection curve is noted down, followed by pointing out the subsequent mode of failure. Further, uniaxial tensile and flatwise compression tests were carried out in accordance with ASTM standards; the output of the experimental results is tabulated in Table 2.

**Table 2:** Extracted material properties of glass fibre, carbon fibre, and balsa wood.

Material	Properties	Test standard	Test content	Value
Glass fibre	Tensile	ASTM D3039	Strength (*MPa*)	3100
			Modulus (*GPa*)	76
Carbon fibre	Tensile	ASTM D3039	Strength (*MPa*)	3530
			Modulus (*GPa*)	230
Balsa wood	Flatwise compression	ASTM C365	Strength (*MPa*)	12.67
			Modulus (*GPa*)	3.95
			Density (*kg/m*^*3*^)	150

## 3 Results and discussion

This section provides a detailed discussion of several modes of failure, which may be influenced by several factors, such as varying span length (*l*ʹ), core thickness (*c*ʹ), and skin thickness (*h*ʹ). For the analysis, it is assumed that both the top and bottom skin are made of a solid and perfectly plastic material. Further, the core material is considered to be rigid-perfect plastic. It is also considered that there is a perfect adhesion between the skin and the core material. Moreover, several failure modes of asymmetric balsa-core based fibre composite sandwich beams are carried out analytically as well as experimentally. These failure modes are the key metric for any structural designers and engineers to improve structural integrity and reliability.

**Fig. 4:**
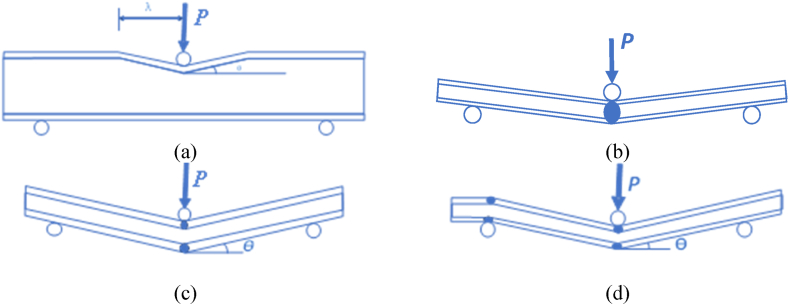
Failure modes of sandwich beams under 3-point bending test [22]: (a) indentation, (b) face yield, (c) core shear-A and (d) core shear-AB.

Among several failure modes in sandwich structures, the most frequently observed modes include indentation, face yield, and core-shear of type A and type AB, as depicted in Fig. 4. Further, the specific modes of failure depend on several factors, such as design considerations, materials used, loading conditions, and the application under which the structure is used. Thus, before analysing the effect of span lengths, core thicknesses, and skin thicknesses experimentally, it becomes crucial to predict the behavior analytically. In view of the above, an analytical model used throughout this study is equated in this section.

In order to begin the analysis, the centre point of deflection in the structure to the unloaded area has been considered to be λ, and deflection of the structure from the skin deviates at an angle θ, as depicted in Fig. 4(a). Since the structure has undergone indentation failure, therefore, considering upper bound theory [30], we get equation (1) [28]:

(1) $$P=\frac{\sigma_{f}bh\prime^{2}}{\lambda }+b\lambda \sigma_{c}$$

where *P*, *b*, *σ*_*f*_, and *σ*_*c*_ are load applied, width of the specimen, yield strength of skin, and compressive strength of the core. The peak point obtained from equation (1) is the critical load under which the structure enters into the indentation failure (*P*_*ind*_) [30]. Since the structure is asymmetric, equation (1) is provided by assuming that the back face has a rigid foundation. Therefore, to analyse the indentation failure more accurately for our structure, it becomes necessary to incorporate the back face materials in equation (1). Thus, while analysing the specimen where the load is projected to CL face, the back face value of 1 of GL has to be considered as equated in the modified equation (3). Likewise, when the load is projected in GL face, the back face value of 1.8 of CL is considered equated in the modified equation (2). Herein, we modified the theoretical values of CL mathematically, i.e., fit the points of CL with a direct proportion function, and the generated proportionate coefficient is 1.8.

(2) $${\bar{P}}_{in-cl}=3.6\bar{c}\bar{h}\sqrt{\frac{2\beta \bar{\sigma }}{1+\beta }}$$

(3) $$\text{and},{\bar{P}}_{in-gl}=2\bar{c}\bar{h}\sqrt{\frac{2\beta \bar{\sigma }}{1+\beta }}$$

where, $\bar{P}=P/\left[bl^{\prime }\left(\sigma_{f}+\sigma_{b}\right)/2\right]$, $\bar{\sigma }=2\sigma_{c}/\left(\sigma_{f}+\sigma_{c}\right)$, $\bar{c}=c'/l'$, $\bar{h}=h'/c'$, and $\beta =\sigma_{f}/\sigma_{b}$, respectively.

Secondly, the analysis of face yield is equally important before analysing the behavior of the fabricated specimen. Since the skin of the structure deforms under bending or loading conditions, as depicted in Fig. 4(b). Thus, following the upper bound theory stated by Jones et al. [30], the critical face yield failure load is derived to be:

(4) $$P_{FY}=4M_{P}/l^{\prime }$$

where, M_P_ stands for the bending moment of the asymmetric sandwich structure. Further, the non-dimensional predicted face yield failure mode is obtained [28]:

(5) $${\bar{P}}_{FY}=\left\{\begin{array}{l} \frac{{\bar{c}}^{2}\left\{4{\bar{h}}^{2}\left[1+\beta \left(4-\beta \right)\right]+4\bar{h}\left[4\beta +\bar{\sigma }\left(1-\beta^{2}\right)\right]+\bar{\sigma }\left(1+\beta \right)\left[4-\bar{\sigma }\left(1+\beta \right)\right]\right\}}{2\left(1+\beta \right)},\delta <-1\\ {\bar{c}}^{2}\left\{4{\bar{h}}^{2}\left[1-\frac{{\left(\beta -1\right)}^{2}}{\bar{\sigma }{\left(\beta +1\right)}^{2}}\right]+4\bar{h}+\bar{\sigma }\right\},-1\leq \delta \leq 1\\ \frac{{\bar{c}}^{2}\left\{4{\bar{h}}^{2}\left[\beta \left(4+\beta \right)-1\right]+4\bar{h}\left[4\beta +\bar{\sigma }\left(\beta^{2}-1\right)\right]+\bar{\sigma }\left(1+\beta \right)\left[4\beta -\bar{\sigma }\left(1+\beta \right)\right]\right\}}{2\beta \left(1+\beta \right)},\delta >1 \end{array}\right\}$$

$$where\;\delta \;is\;\frac{2\bar{h}\left(\beta -1\right)}{\bar{\sigma }\left(\beta +1\right)}\text{.}$$

The equation (5) is derived assuming that the materials used are rigid and perfectly plastic. However, in our analysis, carbon and glass fibres are used to prepare the specimen. The materials used in the specimen have shown a significant increase in the load carrying capacity, which causes a continuous increase in the load even after the face yield failure (*discussed in the later section*). Further, it is difficult to find out the fitting parameter used to determine the accurate face yield failure under CL or GL loading. Therefore, an alternative approach has been employed to verify our results, where we have extracted the experimental values from the load-deflection curve for face-yield failure and also numerically analysed the value from equation (5). Despite some uncertainties associated with the application of this approach, it is possible to control and regulate the level of uncertainty involved [28] (*analysed in the later section*).

Finally, the importance of core material is of utmost importance for analysing any structure. Since we have used balsa wood as a core material as depicted in Fig. 4(c) for core shear type-A and Fig. 4(d) for core shear type-AB, which is lightweight compared to other materials used to date. Thus, to verify the sanity of the experimental behavior, the analytical model plays a significant role to determine the performance shift from the theoretical perspective. For the analysis, the same upper bound theory [30] has been used to analyse the core-shear failure, as equated in equation (6) [28]:

(6) $$P_{A}=\frac{\sigma_{f}+\sigma_{b}}{2}*bh\prime^{2}+2\tau_{c}bc^{\prime }\left(\frac{2{l_{H}}^{\prime }}{l^{\prime }}+1\right)$$

where *P*_*A*_ and $\tau_{c}$ stands for critical collapse load and shear strength of balsa wood core when $\tau_{c}$ ≈ 2*σ*_*c*_*/*3. The equation (6) is derived assuming that the core is rigid and perfectly plastic. Since we have used balsa wood as a core material, therefore, to predict an accurate shift in structural behavior, equation (6) is modified considering the material properties of balsa wood. The modified non-dimensional equation becomes [28]:

(7) $${\bar{P}}_{A}=2{\bar{c}}^{\prime }\left[{\bar{c}}^{\prime }\bar{h}\prime^{2}+\tau \left(2{\bar{l}}^{\prime }+1\right)\right],\text{for}\;\text{core}-\text{shear}\;\text{of}\;\text{type}\;A$$

where, $\bar{\tau }=2\tau_{c}/\left(\sigma_{f}+\sigma_{b}\right)$ and ${\bar{l}}^{\prime }=\frac{l_{H}^{\prime }}{l^{\prime }}$ Likewise, when the structure enters into another type of core-shear failure, type AB, the equation becomes:

(8) $${\bar{P}}_{AB}={\bar{c}}^{\prime }\left[3{\bar{c}}^{\prime }{\bar{h}}^{2}+2\bar{\tau }\left({\bar{l}}^{\prime }+1\right)\right]$$

The experimental results are extracted once the analytical models of frequently observed failures are determined. Later, the measured and experimental values are compared to get the deviation from the predicted value in the next section.

### 3.1 Effect of span length

The span length of the structure is attributed to the distance between two lower rollers where the specimen is fixed, as shown in Fig. 1. For the analysis, span length of 272, 124, and 107 mm have been considered, whereas, core thickness and skin thickness are kept constant at 15 mm and 1.15 mm, respectively. Since the specimen under test has been subjected to bending loads. As a result, the bending moment of the specimen varies. The bending moment led to the flexural failure, such as indentation, face yield, and core shear. The structure fails the bending test due to excessive stress, depending on their material and mechanical properties.

**Fig. 5:**
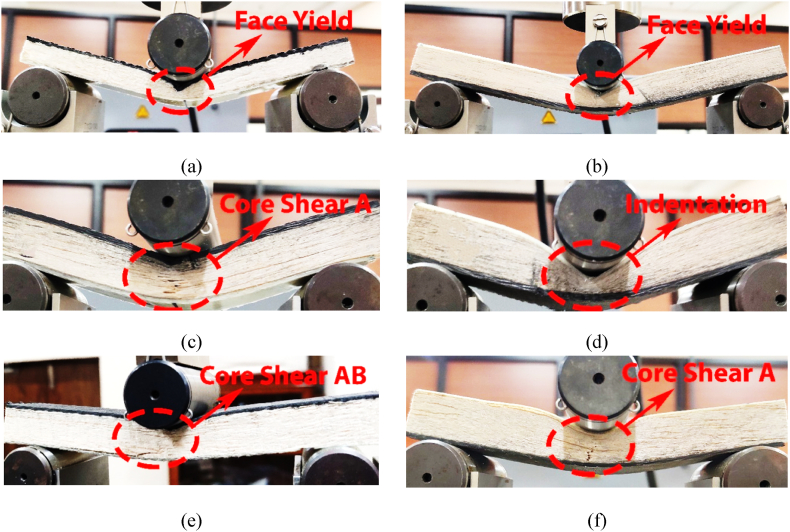
Effect of span length for *hʹ* = 1.15 mm and *cʹ* = 15 mm for: (a) CL-01, (b) GL-02, (c) CL-06, (d) GL-03, (e) CL-05, and (f) GL-04.

**Fig. 6:**
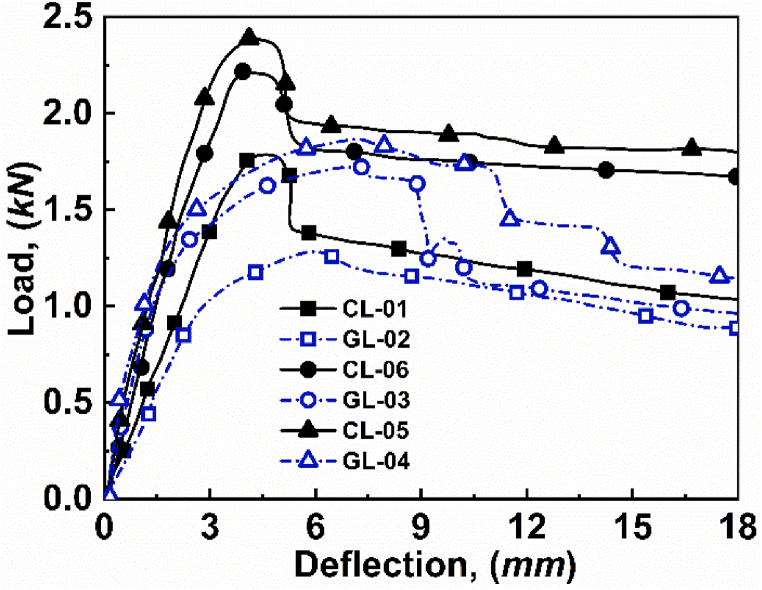
Effect of span length on load-deflection curve for *hʹ* = 1.15 mm and *cʹ* = 15 mm.

The cross-section view of varying span lengths while performing a 3-point bending test on several specimens is depicted in Fig. 5. It is observed that with decreasing span length from 272 to 107 mm, the load distribution of the sandwich structure changes. In addition, the results of Fig. 5 also show that the maximum bending moment appeared to be at the centre of the structure. In contrast, the magnitude of the structural bending moment decreases towards the support. As a consequence, the longer span length is highly susceptible to entering the face yield than to entering either indentation or core shear, as depicted in Fig. 5(a) and (b). It is also observed that longer span lengths have independence in the type of failure modes on the loading faces, i.e., either CL face or GL face. Moreover, the most extended span length results in increased deflection, which induces comparatively higher stress in the centre than the shorter span lengths [16]. As a result, longer span length fails with a lighter load to the structure, where the load on CL face fails initially at 2393 *N* and GL at 1284 *N* loads, as depicted in Fig. 6.

With decreasing span length, the deflection of the structure reduces, which led to increasing shear stress at the centre of the structure [31]. If the shear force exceeds the critical limit, then instead of face yield, the structure experiences core shear failure. Apart from the shear force, the face loading material also plays a significant role, where the higher young's modulus of carbon fibre provides extra strength to the material [32]. Thus, for span length of 124 mm, the CL face loading shows core-shear A failure while GL face loading shows indentation. This different failure mode is due to the strength and flexibility of the material. Since, glass fibre is highly flexible then carbon fibre, therefore GL face loading shows indentation while CL face load shows core-shear A, as depicted in Fig. 5(c) and (d), respectively. Finally, when span length is further reduced to 107 mm, then the shear stress at the centre of the structure dominates, which surpass the critical flexible limit of glass fibre. Therefore, both CL face and GL face loading shows core-shear failures of type AB (Fig. 5(e)) and type A (Fig. 5(f)). As we know, with reducing span length, the load carrying capacity of the structure increases, i.e., 272 mm *l*ʹ can withstand 1785 *N* for CL and 1284 *N* for GL, 124 mm *l*ʹ can withstand 2207 *N* for CL and 1729 *N* for GL, and 107 mm *lʹ* can withstand 2393 *N* for CL and 1868 *N* for GL, respectively, as shown in Fig. 6.

**Fig. 7:**
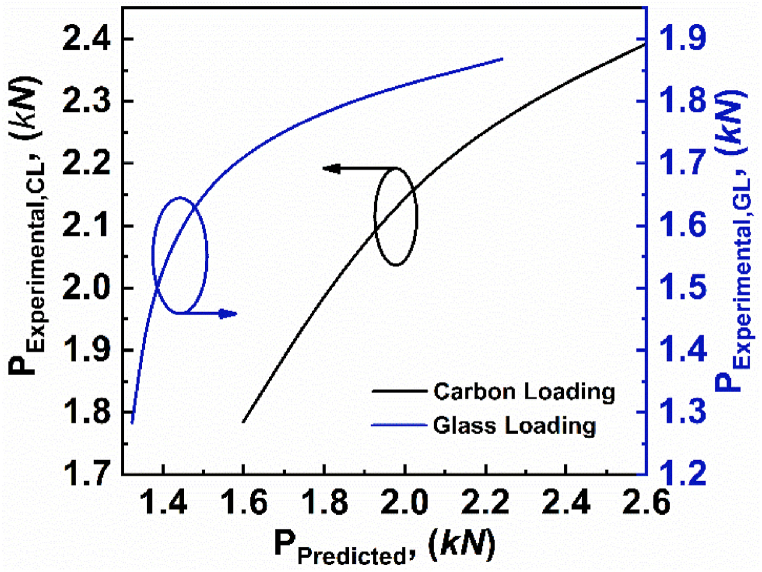
Experimental critical load as a function of predicted critical load for varying span lengths.

In order to verify the experimental results, the analytical models stated in equations (2), (4), (5), (6), (7), (8) are used. The predicted value and the experimental values for varying span lengths are tabulated in Table 3. Further, the experimental critical load as a function of predicted critical load for varying span lengths, are depicted in Fig. 7. The comparison graph shows that the experimental critical load follows the predicted critical load with some minor errors. The deviation between experimental value and predicted value of load can be attributed to an analytical model that assumes the rear face to be stiff, and a glass fiber loading face (GL) specimen comes closer than a carbon fiber loading face (CL) specimen.

**Table 3:** Critical load values of varying span lengths for experimental and predicted loads.

Specimen	h' (*mm)*	c' (*mm*)	l' (*mm*)	Failure Mode	P_Eperimental_ (*N*)	P_Predicted_ (*N*)	Error (%)
CL-01	1.15	15	272	FY	1785	1599	10.42
GL-02	1.15	15	272	FY	1284	1323	3.03
CL-06	1.15	15	124	CS-A	2207	2007	9.06
GL-03	1.15	15	124	IND	1729	1393	19.43
CL-05	1.15	15	107	CS-AB	2393	2601	8.69
GL-04	1.15	15	107	CS-A	1868	2241	19.96

### 3.2 Effect of varying skin thickness

**Fig. 8:**
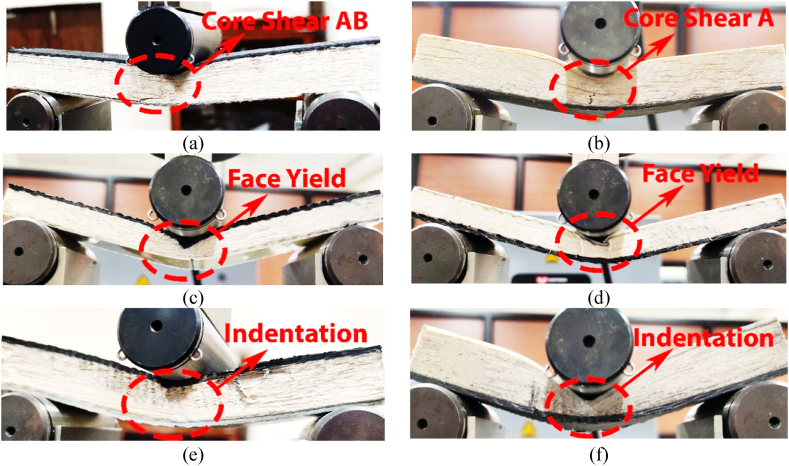
Effect of skin thickness for *lʹ* = 107 mm and *cʹ* = 15 mm for: (a) CL-05, (b) GL-04, (c) CL-14, (d) GL-13 (e) CL-18, and (f) GL-17.

The CFRP and GFRP materials are used for the skin thickness. Since the CFRP and GFRP material possesses several advantages over the most frequently used metals for sandwich structures [28]. Further, CFRP materials exhibit higher mechanical properties, such as higher modulus of elasticity and tensile strength, than that of GFRP [32]. In addition, CFRP is lighter and has lower thermal conductivity compared to GFRP. These properties make the structure more effective in the application where heat transfer control is important. We consider the material thickness as a factor in determining the load carrying capacities on both sides of the structure. Hence, it is imperative to ensure proper maintenance of the application in which the structures are intended to be utilized. In addition, increasing the skin thickness generally enhances the flexural strength of the structure, as illustrated in Fig. 8. The reason for increasing flexural strength is for better resistance towards the bending moment that results in distributed load over a larger surface area, which in turn reduces the stress on the core of the structure. Thus, increasing skin thickness increases the load carrying capacity, as depicted in Fig. 8.

The study examines the effect of skin thickness on the mechanical properties of carbon fibre reinforced polymer (CFRP) and glass fibre reinforced polymer (GFRP) materials. Specifically, specimens with thicknesses of 1.15, 1.4, and 1.85 mm were used for CFRP and GFRP materials. In addition, other parameters, such as a span length of 107 mm and core thickness of 15 mm, are kept constant throughout the analysis. It is observed from Fig. 8 that when skin thickness increases, then the load is distributed throughout the specimen. As a result, with increasing skin thicknesses, instead of core-shear as appeared for 1.15 mm CL or GL face loading (Fig. 8(a) and (b)), the specimen shows face yield for 1.4 mm CL or GL face loading (Fig. 8(c) and (d)), then indentation failures for skin thickness of 1.85 mm in case of CL or GL face loadings, as depicted in Fig. 8(e) and (f), respectively.

**Fig. 9:**
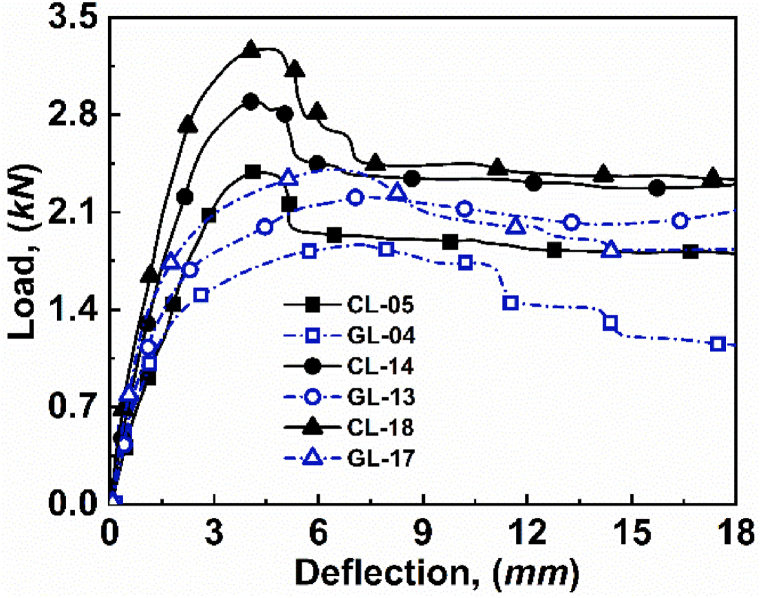
Effect of skin thickness on load-deflection curve for *lʹ* = 107 mm and cʹ = 15 mm*.*

The thicker skin distributes the shear forces and contributes to the higher moment of inertia throughout the specimen, thus reducing the stress concentration and deflection at the centre, as depicted in Fig. 9. It is observed that the smaller skin thickness of 1.15 mm for CL and GL face load has load carrying capacities of 2391 *N* and 1869 *N*, respectively. However, increased skin thickness to 1.4 and 1.85 mm has shown 2890 N and 3277 N for CL face and 2220 *N* and 2413 *N* for GL face loading, respectively.

In order to verify the experimental results of varying skin thicknesses, the analytical models stated in equations (2), (4), (5), (6), (7), (8) are used. The predicted value and the experimental values for varying skin thicknesses are tabulated in Table 4.

**Table 4:** Critical load values of varying skin thicknesses for experimental and predicted loads.

Specimen	h' (*mm)*	c' (*mm*)	l' (*mm*)	Failure Mode	P_Experimental_ (*N*)	P_Predicted_ (*N*)	Error (%)
CL-05	1.15	15	107	CS-AB	2391	2601	08.78
GL-04	1.15	15	107	CS-A	1869	1512	19.10
CL-14	1.4	15	107	FY	2890	2703	06.47
GL-13	1.4	15	107	FY	2220	2331	05.00
CL-18	1.85	15	107	IND	3277	3591	09.58
GL-17	1.85	15	107	IND	2413	2736	13.38

**Fig. 10:**
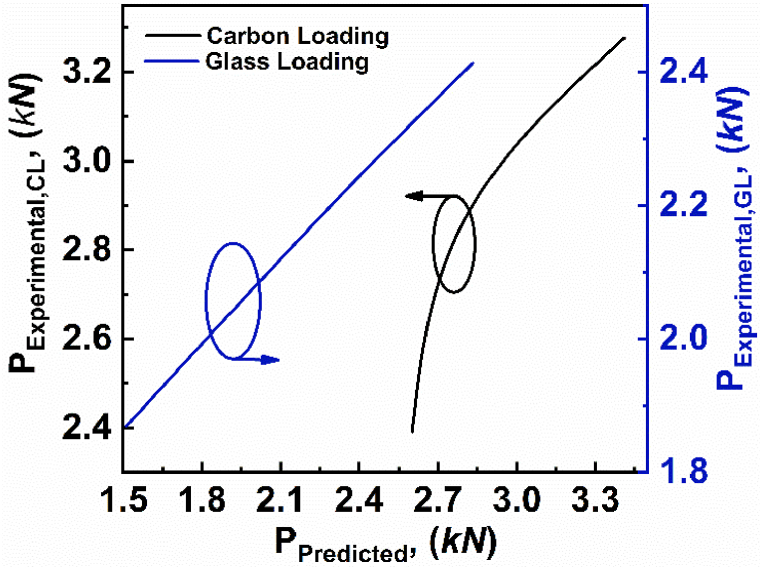
Experimental critical load as a function of predicted critical load for varying skin thicknesses.

Further, the experimental critical load as a function of predicted critical load for varying skin thicknesses, are depicted in Fig. 10. The deviation between experimental value and predicted value of load could be caused by the analytical model, which incorporates assumptions and approximations to depict the behavior of the asymmetric composite sandwich structures. The comparison graph shows that the experimental critical load follows the predicted critical load with some minor errors.

### 3.3 Effect of varying core thickness

**Fig. 11:**
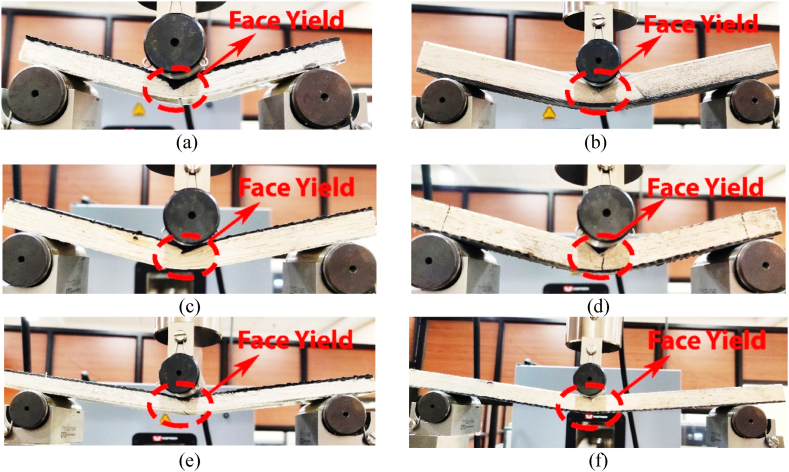
Effect of core thickness for *lʹ* = 272 mm and *hʹ* = 1.15 mm for: (a) CL-01, (b) GL-02, (c) CL-10, (d) GL-09, (e) CL-12, and (f) GL-11.

The core thickness has a significant effect on failure modes under 3-point bending. The core thickness affects the load distribution and stiffness within the structure [33]. It is understood that the thicker core provides better resistance toward the shear stress and bending of the structure, which result in even distribution of the load over the face sheets. Thus, it reduces the likelihood of core-shear and localized plastic deformation [34]. In addition, it is also crucial to optimize the core thickness because a thicker core can add extra weight and increases the cost of the structure. Therefore, to provide a balance between the cost, weight, load distribution, and stiffness of the structure, an optimum core needs to be struck. In view of these, balsa wood as a core material has been chosen. Its effect on the asymmetric structure for varying core of 15 mm, 12 mm, and 9 mm have been selected for the comparative analysis, keeping *h*ʹ as 1.15 mm and *lʹ* as 272 mm, constant, as depicted in Fig. 11.

It is observed that with increasing core thicknesses, the load carrying capacity of the structure increases, as depicted in Fig. 11. The increased thicknesses reduce the chances of core-shear failure mode when either CFRP or GFRP materials are used as face materials because the skin faces, particularly CFRP, distribute the shear forces over a larger area that results in enhanced core shear strength and reduces the chances of core shear failure. The statement is verified in Fig. 11, where three different cores have been used: 15 mm, 12 mm, and 9 mm, respectively. Although the core size varies, the CFRP and GFRP material, in combination, reduces the chances of core shear in the structure. As a result, with increasing core thicknesses, the same failure modes, i.e., face yield, appeared for core thicknesses of 15 mm CL or GL face loading (Fig. 11(a) and (b)), for 12 mm CL or GL face loading (Fig. 11(c) and (d)), and for 09 mm CL or GL face loadings, as depicted in Fig. 11(e) and (f), respectively.

**Fig. 12:**
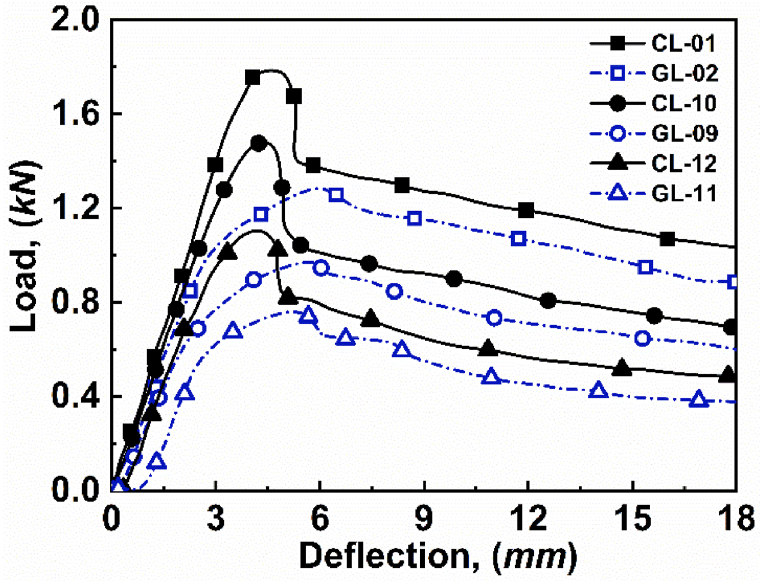
Effect of core thickness on load-deflection curve for *lʹ* = 272 mm and *hʹ* = 1.15 mm.

The effect is visualized in Fig. 12, where the core size varies from 15 mm to 9 mm, which results in decreasing the load carrying capacities. Yet, the proposed asymmetric sandwich structure does not let the structure enter into core-shear failure mode. This advantage of CFRP and GFRP in combination can pass to the application where higher strength is required at a low cost.

In order to verify the experimental results of varying core thicknesses, the analytical models stated in equations (2), (4), (5), (6), (7), (8) are used. The predicted value and the experimental values for varying core thicknesses are tabulated in Table 5.

**Table 5:** Critical load values of varying skin thicknesses for experimental and predicted loads.

Specimen	h' (*mm)*	c' (*mm*)	l' (*mm*)	Failure Mode	P_Experimental_ (*N*)	P_Predicted_ (*N*)	Error (%)
CL-01	1.15	15	272	FY	1785	1863	04.36
GL-02	1.15	15	272	FY	1284	1443	12.38
CL-10	1.15	12	272	FY	1489	1233	17.19
GL-09	1.15	12	272	FY	981	1233	25.68
CL-12	1.15	09	272	FY	1108	922	16.78
GL-11	1.15	09	272	FY	766	840	09.66

**Fig. 13:**
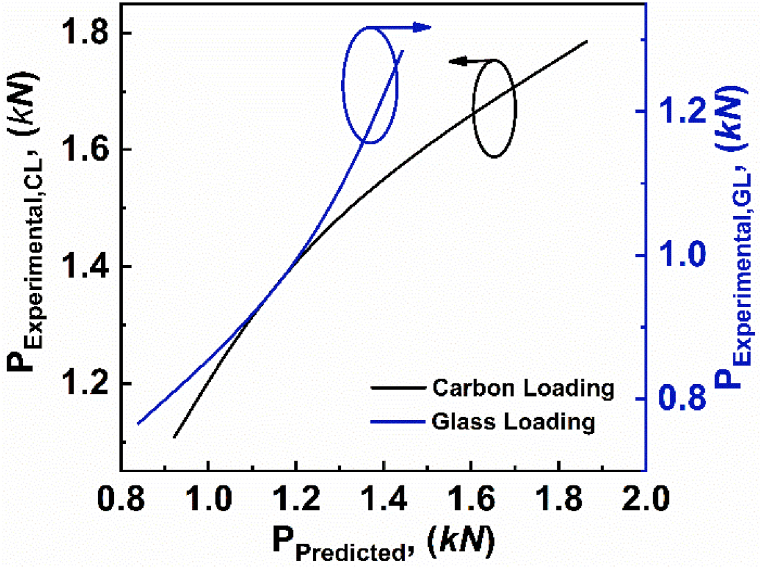
Experimental critical load as a function of predicted critical load for varying core thicknesses.

Further, the experimental critical load as a function of predicted critical load for varying core thicknesses, are depicted in Fig. 13. The deviation between experimental value and predicted value of load could be attributed to the inherent simplifications and assumptions inherent in the analytical model used to characterize the behavior of asymmetric composite sandwich structures. The comparison graph shows that the experimental critical load follows the predicted critical load with some minor errors.

### 3.4 Benchmarking of the structure against already available reports on asymmetric structures

In this section, the behavior of the proposed structure has been compared with several published research in terms of load-carrying capacity (LC capacity), cost effectiveness, and nature of the structure. It is observed that most of the previously published reports are costly as well as corrosive in nature. Wang et al. [28] and Zhang et al. [25] have reported a structure with load carrying capacity of 5782 *N* and 6169 *N*. However, the structure is costly and corrosive in nature. On the other hand, Hoto et al. [38] have reported an asymmetric sandwich structure with basalt and flax fibre as skin material and corc board as the core. Although the structure has economical nature, the load carrying capacity is far less than the previously reported literature. Thus, this work has been carried out to enhance the load carrying capacity considering the nature and cost of the structure to as low as possible. The benchmarking of the proposed structure against previously available reports on asymmetric structures is tabulated in Table 6.

**Table 6:** Benchmarking of the proposed Asymmetric structure.

References	Structure	Skin material	Core material	LC Capacity (*N*)	Cost per unit load capacity (N/$)	Nature of the structure
[28]	Asymmetric	Aluminium and Steel	Aluminium foam	5782	0.25	Corrosive
[35]	Symmetric	GFRP	Aluminium honeycomb	2073.5	0.1	Corrosive
[25]	Asymmetric	Al alloy	Aluminium foam	6169	0.27	Corrosive
[36]	Symmetric	Al alloy	Aluminium foam	2550	0.11	Corrosive
[37]	Symmetric	GFRP	Aluminium foam	1860	0.09	Corrosive
[38]	Asymmetric	Basalt and flax fibre	Corc board	340	0.17	Non-corrosive
**This work**	Asymmetric	CFRP and GFRP	Balsa wood	3277	0.16	Non-corrosive

## 4 Conclusion

The analytical and experimental analysis demonstrates that the asymmetric sandwich structure has more efficient weight distribution and design flexibility compared to the symmetric structures. In addition, composite materials (i.e., carbon and glass fibres) with different densities and properties on top and bottom faces offer several advantages, such as high strength-to-weight ratio, corrosion resistance, fatigue resistance, thermal insulation, and cost effectiveness. This study uses balsa wood as the core material, which offers excellent shear strength, lightweight, and high energy absorption capabilities. It is observed that the carbon fibre loading shows a maximum load carrying capacity of 3277 *N*, which is significant for such a lightweight category structure. After analytical and experimental analysis, it is noted that composites not only offer higher strength and stiffness, but they can also distribute the load throughout the surface so that the core does not experience excess shear stress. In order to analyse such properties, the effect of varying span lengths from 272 to 107 mm, skin thicknesses from 1.15 to 1.85 mm, and core thicknesses from 15 to 9 mm, are taken into consideration. The 3-point bending test on varying span lengths shows that even with a longer span length structure, the carbon fibre evenly distributes the load throughout the surface, and the balsa core strengthens the structure. Secondly, the analysis of varying skin thicknesses reveals that the minimum possible thickness of carbon fibre composite that offers extraordinary strength toward the load carrying capacity. However, with increasing skin thickness, instead of core-shear failure, the structure provides better resistance towards the projected load and allows the structure to enter core-shear failure. Thirdly, the varying balsa wood core with optimum skin thickness and span lengths provides very high load carrying capacity and has shown only one type of failure, i.e., face yield failure. Finally, benchmarking the structure against several published works has demonstrated several advantages over already published work. In a nutshell, the proposed asymmetric carbon and glass fibre composites-based sandwich structure with balsa wood as a core has the potential to replace the already existing sandwich structures in industries, such as applications in aerospace, automotive, marine, and renewable energy sectors.

This study would be interesting to investigate the possible variations in findings that may arise from using alternative composite materials, such as aramid fiber and basalt fiber, as face material. Additionally, the research currently overlooks the influence of environmental aspects, such as temperature and humidity, on the behavior of the sandwich beam. The broader applicability of the findings could be better understood through the incorporation of ambient factor analysis.

## Data availability

The data that support the findings of this study are available on request from the corresponding author.

## CRediT authorship contribution statement

**Prahlad Kumar:** Writing – review & editing, Writing – original draft, Visualization, Validation, Software, Resources, Methodology, Investigation, Funding acquisition, Formal analysis, Data curation, Conceptualization. **K. Priya Ajit:** Writing – review & editing, Validation, Supervision, Resources, Project administration, Investigation, Funding acquisition, Conceptualization. **Jayant Prakash Varun:** Writing – review & editing, Validation, Resources, Project administration.

## Declaration of competing interest

The authors declare that they have no known competing financial interests or personal relationships that could have appeared to influence the work reported in this paper.
